# Validation of a Multimodal Wearable Device Integrating EMG and IMU Sensors for Monitoring Upper Limb Function During Tooth Brushing Activities of Daily Living

**DOI:** 10.3390/s26020510

**Published:** 2026-01-12

**Authors:** Patrícia Santos, Filipa Marquês, Carla Quintão, Cláudia Quaresma

**Affiliations:** 1 Laboratory for Instrumentation, Biomedical Engineering and Radiation Physics (LIBPhys), NOVA School of Science and Technology, NOVA University of Lisbon, 2829-516 Caparica, Portugal; cmquintao@fct.unl.pt (C.Q.); q.claudia@fct.unl.pt (C.Q.); 2 Associated Laboratory in Translation and Innovation Towards Global Health (REAL), NOVA School of Science & Technology, NOVA University of Lisbon, 2829-516 Caparica, Portugal; 3 Physics Department, NOVA School of Science and Technology, NOVA University of Lisbon, 2829-516 Caparica, Portugal; fmv.marques@campus.fct.unl.pt; 4 Health Department, Superior School of Health, Polytechnic Institute of Beja, 7800-111 Beja, Portugal

**Keywords:** wearable sensors, electromyography, inertial measurement units, upper limb, activities of daily living, optoelectronic motion capture system, biomechanics

## Abstract

Analyzing the dynamics of muscle activation patterns and joint range of motion is essential to understanding human movement during complex tasks such as tooth brushing Activities of Daily Living (ADLs). In individuals with neuromotor impairments, accurate assessment of upper limb motor patterns plays a critical role in rehabilitation, supporting the identification of compensatory strategies and informing clinical interventions. This study presents the validation of a previously developed novel, low-cost, wearable, and portable multimodal prototype that integrates inertial measurement units (IMU) and surface electromyography (sEMG) sensors into a single device. The system enables bilateral monitoring of arm segment kinematics and muscle activation amplitudes from six major agonist muscles during ADLs. Eleven healthy participants performed a functional task, tooth brushing, while wearing the prototype. The recorded data were compared with two established gold-standard systems, Qualisys^®^ motion capture system and Biosignalsplux^®^, for validation of kinematic and electrophysiological measurements, respectively. This study provides technical insights into the device’s architecture. The developed system demonstrates potential for clinical and research applications, particularly for monitoring upper limb function and evaluating rehabilitation outcomes in populations with neurological disorders.

## 1. Introduction

Technology plays a critical role in the analysis of movement patterns, providing the level of precision that conventional scale-based assessment tools inherently lack [1]. Examples of this include conventional clinical methods such as manual goniometry and manual muscle testing, both of which present important limitations. Studies highlight the inconsistent intra- and inter-rater agreement associated with goniometric assessments [2] and demonstrate that manual muscle testing is inherently subjective, with outcomes influenced by factors such as the examiner’s clinical experience, correct application of the procedure, and proper patient positioning [3].

The use of technologies in movement analysis continues to grow, with advancements in wearable devices and motion capture systems enabling the collection of increasingly precise biomechanical data [4,5], particularly in the analysis of complex movement patterns, such as those performed during ADLs. Upper-body kinematic measures are widely used in several fields, such as rehabilitation [6,7], to describe normal and pathological motion of different body segments, including the trunk, head, and arms [8].

There is a growing interest in the research and clinical communities, across different fields of knowledge, in analyzing human movement in naturalistic settings, outside traditional laboratory environments [9], to improve understanding of the biomechanical stress imposed on anatomical structures as a contributing factor to injury. Although the most common motion capture methods rely on laboratory-based marker systems and electromagnetic technologies to obtain highly precise kinematic measurements (within 1–3°) [6], there is increasing interest in the development of wearable sensor technologies that are easy to apply, low-cost, with sufficient accuracy to the needs of clinical practice.

Biosignals such as electromyography also offer valuable advantages, providing in-depth information as an assessment tool in the fields of prevention, rehabilitation monitoring, and training [10]. In recent decades, technological advances have driven the development of new sEMG solutions and enabled their synchronization with other technologies, such as accelerometry, force platforms, and motion capture systems [10].

Within this line of integrated wearable systems combining kinematic and biosignals acquisition, a Knee Angle Measurement (KAM) device was developed that integrates IMU-based knee angle estimation with multichannel sEMG [11]. The system showed excellent performance, with high linearity (R^2^ ≈ 1.00), very low error in controlled tests (full-range error 0.25–0.92°; RMSE 0.05–0.15°), and strong agreement with the Qualisys optical motion-tracking gold standard during gait (cross-correlation > 0.95; RMSE 5.5–8°). Although the sEMG module provided high-quality signals (median SNR 108 dB), it was not validated against a gold-standard reference for muscle activation amplitude, and the device does not allow bilateral simultaneous assessment of upper or lower limbs during functional tasks. Furthermore, although the authors state that the system could be applied to other body segments and activities, the device does not allow bilateral assessment of two limbs simultaneously, whether upper or lower limbs, during functional tasks.

Within the scope of commercially available wearable devices, systematic reviews [12] frequently highlight the Myo Armband [13] and the Delsys Trigno™ Wireless EMG System (Delsys Inc., Natick, MA, USA) [14]. The Myo Armband (Thalmic Labs, Waterloo, ON, Canada) is a low-cost consumer device integrating eight sEMG sensors and a 9-axis IMU, offering easy wireless acquisition but limited by low sampling rates (approximately 200 Hz for sEMG and 50 Hz for IMU) [12], restricted unilateral use, and the inability to be positioned over scapular-region muscles to provide an integrated assessment of shoulder joint kinematics and muscle activation amplitude. In contrast, the Delsys Trigno™ Wireless EMG System (Delsys Inc., Natick, MA, USA) is a high-performance research-grade platform that combines high-fidelity sEMG with 3-axis accelerometry and 3-axis gyroscopy in each wireless sensor [12], ensuring excellent signal quality and flexibility for bilateral and multi-muscle acquisition, albeit at a substantially higher cost and greater operational complexity.

Other studies have combined kinematic and biosignal acquisition, but typically through separate devices operating in parallel—for example, MPU6050-based IMUs connected to a custom DCU for motion tracking and a Wave COMETA wireless multichannel EMG system (Cometa Systems, Milan, Italy) [15]. In this study, the IMU module demonstrated strong validity against the VICON optoelectronic camera system (Vicon Motion Systems, Oxford, UK) gold standard across all rotational components. For the yaw angle, a low mean bias of 0.224° ± 2.1° was observed, with narrow limits of agreement (−3.180° to 3.628°), high concordance (LCC = 0.958), and strong linearity (r^2^ = 0.976). Similarly, the pitch angle showed a low mean bias of 0.139° ± 1.599°, with limits of agreement ranging from −2.397° to 2.676°, high concordance (LCC = 0.908), and strong linearity (r^2^ = 0.924). For the roll angle, the mean bias was −0.864° ± 0.237°, with limits of agreement of −4.474° to 2.745°, accompanied by high concordance (LCC = 0.928) and strong linearity (r^2^ = 0.951). Although the EMG signals displayed consistent activation patterns, they were not compared with a gold-standard reference for activation amplitude.

Although not in an integrated and synchronous form, other devices have been developed and validated for the assessment of kinematic variables, namely using IMU-based systems [8,16,17,18], and biosignals, namely using sEMG-based systems [10,19,20], focus on the premise of creating easy-to-use, low-cost systems that can be applied outside laboratory settings while still providing adequate measurement accuracy [8].

These system characteristics are essential for evaluating kinematic variables and biosignals in individuals with impairments, particularly those resulting from neurological disorders [21,22,23], who perform tasks involving complex movement patterns, such as the analysis of upper limb (UL) neuromotor function. This is exemplified by patients with stroke, in whom hemiparesis of the contralateral UL is the most common deficit, affecting more than 80% of individuals in the acute phase [24]. Such impairment leads to omissions of small actions and alterations in movement sequence and quality during ADLs, as demonstrated in studies on meal preparation [25] and hygiene tasks [26], ADLs with complex movement patterns.

Sensor-based technologies have emerged as valuable tools for the quantitative analysis of movement patterns associated with ADLs [27], providing high-resolution data that surpass the precision of conventional assessment methods, such as qualitative scales and goniometry. This level of analytical precision will enable the monitoring of compensatory movement patterns that arise due to reduced strength, abnormal muscle tone, pain, and other contributing factors [28,29]. Such compensations may be detrimental to the long-term functional outcome of the affected arm, as they often produce inefficient movement strategies that limit progress and hinder recovery of the paretic limb [30,31], thereby supporting the optimization of rehabilitation strategies for stroke survivors [32].

The integration of IMU and sEMG enables the identification of deviations in motor execution across functional tasks. By analyzing these multimodal data, it becomes possible to distinguish between typical and atypical movement patterns, facilitating the early detection of neuromotor dysfunction and supporting rehabilitation strategies [33].

Through our review of the literature, we identified a clear gap in the availability of portable, low-cost and user-friendly devices capable of synchronously collecting kinematic and biosignal data through integrated IMU and sEMG sensors. To address this gap, we aimed to validate a device developed by our research team, which had already demonstrated promising results in preliminary pilot studies [34,35]. Accordingly, we formulated our research central question is whether this low-cost integrated device is sufficiently reliable to assess neuromotor patterns during ADLs.

To answer this question, the present study focused on comparing the measurements obtained from our Wearable IMU–sEMG Device Prototype with those recorded by two established commercial reference systems. The results of this analysis are intended to determine whether the proposed low-cost Wearable IMU–sEMG Device Prototype demonstrates adequate performance or whether further refinements are required.

Data acquisition followed a previously applied protocol [35], in which participants performed the bilateral tooth-brushing task while sEMG signals were collected from the main shoulder muscles [36], pectoralis major (PM), anterior deltoid (AD), middle deltoid (MD), posterior deltoid (PD), upper trapezius (UT), and lower trapezius (LT). IMU signals corresponding to three-dimensional shoulder movements—flexion (F), extension (E), medial rotation (MR), lateral rotation (LR), abduction (ABD), and adduction (ADD), were also recorded. This protocol was executed simultaneously using our integrated IMU–sEMG device and an optoelectronic motion-capture system (Qualisys^®^ optical motion capture system) and subsequently repeated for sEMG acquisition with the Biosignalplux^®^ device on the same muscle set.

## 2. Materials and Methods

### 2.1. Wearable IMU–sEMG Device Prototype and Interface

**Figure 1:**
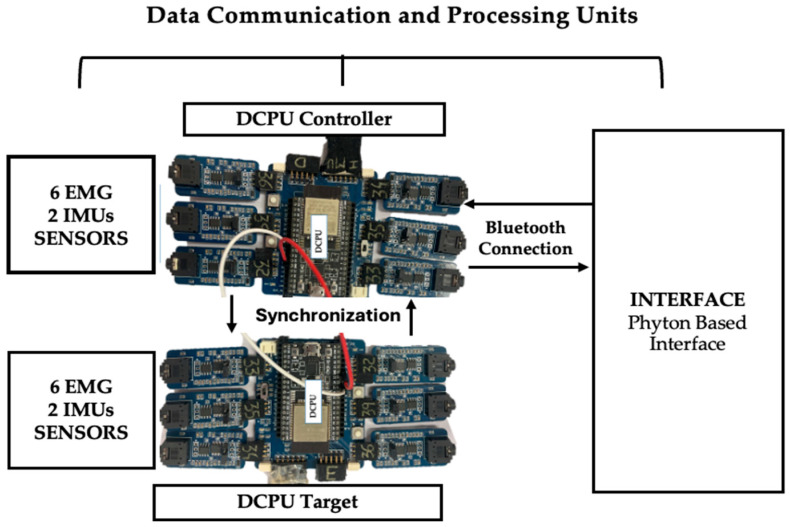
Block diagram illustrating the data communication and processing architecture, where the Master and Slave DCPUs receive input from six EMG sensors and two IMU sensors each, with the Master handling synchronization and Bluetooth transmission to a Python (version 3.13; Python Software Foundation, Wilmington, DE, USA) based interface.

This study used a prototype device consisting of a portable, low-cost data acquisition system, supported by hardware and software developed to acquire, communicate, and visualize EMG and IMU data, for kinematic and electrophysiological analysis [34] (Figure 1). The electronic system, developed by WallySci (Neu-riot Technologies LLP, Bengaluru India), consists of two Data Communication and Processing Units (DCPUs) built around ESP32-WROOM-32D microcontrollers (Espressif Systems, Shanghai, China). Each DCPU connects via I2C to six EMG sensors and one 9DoF IMU and wirelessly transmits the acquired data to the interface via Bluetooth.

The data acquisition is coordinated between two microcontrollers, designated as the Controller and the Target. The Controller acts as the synchronization master, receiving start commands via Bluetooth from the graphical interface and generating a short synchronization pulse through a dedicated GPIO pin. The Target is configured to receive this synchronization signal via an external interrupt and initiates data acquisition upon detection of the rising edge. This allows both boards to begin data collection in a coordinated manner, ensuring temporal alignment of the acquired EMG and IMU signals.

In this way, two microcontrollers (Controller and Target) were programmed in Arduino IDE and connected via Bluetooth to each other and to the graphical interface. Upon receiving command 1, the Controller sent a sync pulse to the Target to align data acquisition. EMG signals were sampled at 1000 Hz and IMU data at 100 Hz. The I2C bus and IMU were initialized with Wire.begin() and imu.begin(), and both microcontrollers continuously monitored data availability, retrieving quaternions via imu.dataAvailable() and converting them to Euler angles (yaw, pitch, roll).

A Python-based interface was developed for real-time visualization and interaction with the portable device, providing connection feedback, start/stop controls, and customizable channel selection. It allows users to view individual sensor data for each limb, input patient information, and display muscle activity from six muscles alongside roll, pitch, and yaw angles in color-coded graphs. Designed for usability, the interface enhances data accessibility and supports more effective analysis and decision-making.

### 2.2. Qualisys^®^ Optical Motion Capture System

As the gold-standard equipment for kinematic data acquisition, a Qualisys^®^ optical motion-capture system (Qualisys^®^, Gothenburg, Sweden) was used, consisting of four Miqus infrared cameras that tracked the trajectories of reflective markers placed on specific anatomical landmarks, in conjunction with the Qualisys Track Manager (QTM) software (version 2025; Qualisys AB, Gothenburg, Sweden) for data acquisition and preprocessing [37].

### 2.3. Biosignalsplux^®^

As the gold-standard equipment for electromyographic signal acquisition, the Biosignalsplux system was used. This physiological data acquisition platform provides high-precision EMG recording and offers 8-channel configurations [38]. In this study, two 8-channel Biosignalsplux^®^ kits (PLUX Wireless Biosignals, Lisbon, Portugal) were used, one per UL, each connected to six EMG sensors, corresponding to the analyzed muscles, and an additional accelerometer sensor placed on the right upper limb to generate a synchronization trigger through an abrupt increase in acceleration amplitude. Data were acquired (sample rate of 1000 Hz) and visualized in real time using OpenSignals (r)evolution^®^ v2.2.5, which communicates with the devices via Bluetooth Low Energy and exports recordings in .txt format for analysis.

### 2.4. Subject Recruitment

An initial sample of 15 healthy adults was recruited, two participants were excluded due to Bluetooth connectivity issues and two due to reflective marker misplacement that prevented kinematic preprocessing in QTM, resulting in a final sample of 11 individuals (5 females, 6 males; all right-handed; mean age 29.4 ± 8.3 years; mean height 171 ± 5 cm). Inclusion criteria required participants to be ≥18 years old with no self-reported musculoskeletal, neurological, or cognitive conditions affecting upper limb motor control. All participants provided written informed consent, and anonymity was ensured. The study followed the Declaration of Helsinki and received ethical approval from NOVA’s Faculty of Science and Technology (CE_FCT_002_2022).

### 2.5. Experimental Setup and Protocol

**Figure 2:**
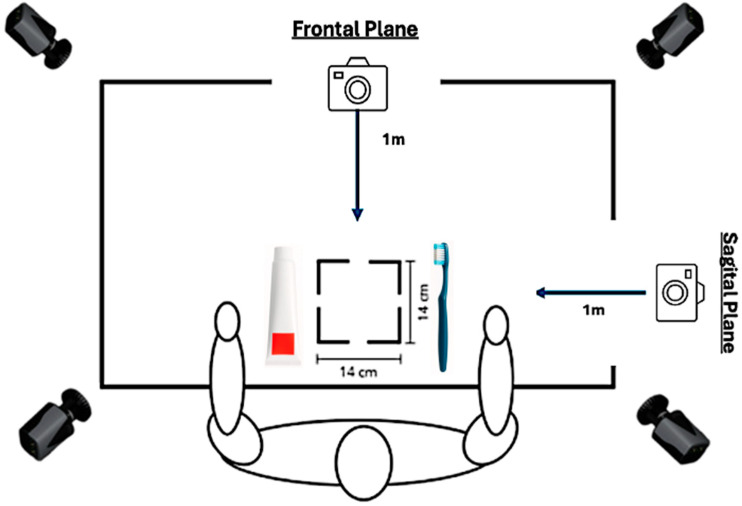
Schematic representation of the laboratory set-up used for the acquisition of kinematic and EMG data during the tooth-brushing AD.

A protocol adapted from previous studies [34,35,39,40] was used for the tooth brushing ADLs. Participants were seated in the same position described in those studies. A napkin was placed 3 cm from the edge of the table and aligned with the participant’s midline, with the toothpaste positioned on the non-dominant side and the toothbrush on the dominant side. The task was first performed at a comfortable, self-selected speed [41], and five repetitions were completed with a 5 s rest interval to minimize fatigue. Two video cameras recording at 30 frames/s (coronal and sagittal planes) were used to identify and segment the ADL cycles (Figure 2).

**Figure 3:**
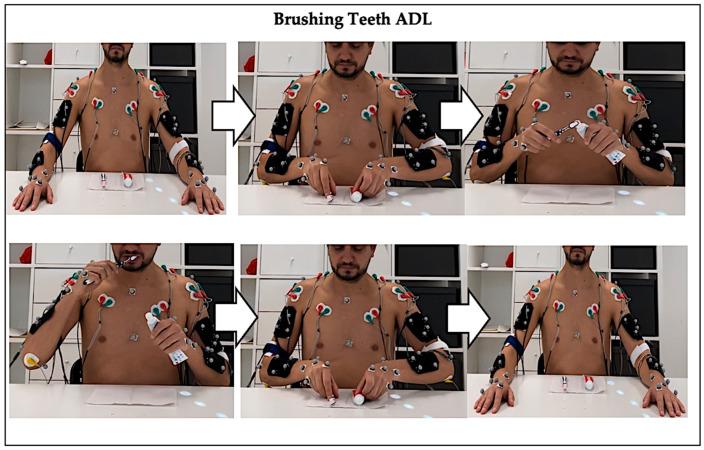
Graphical representation of the brushing teeth ADL.

**Figure 4:**
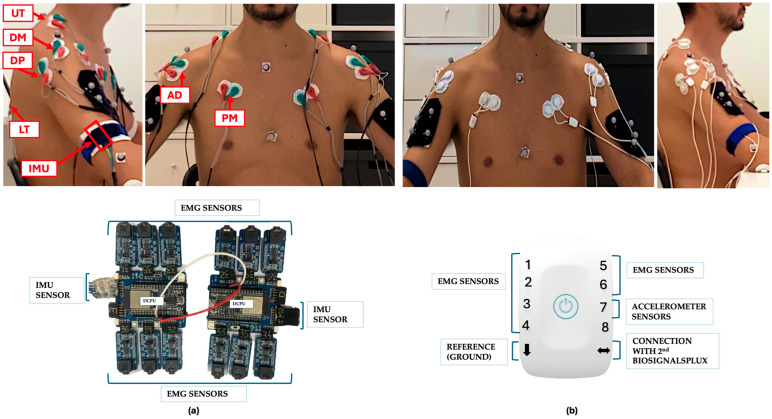
Experimental setup for EMG electrode placement and data acquisition. (**a**) Data acquisition using the wearable IMU–sEMG prototype device, illustrating the placement of EMG electrodes during simultaneous recording with the Qualisys^®^ optical motion-capture system. (**b**) EMG-only data acquisition using the BiosignalsPlux^®^ reference system, with the same EMG electrodes connected to the reference device after disconnecting the prototype cables, ensuring identical electrode placement for both systems. EMG signals were recorded from the pectoralis major (PM), anterior deltoid (AD), middle deltoid (MD), posterior deltoid (PD), upper trapezius (UT), and lower trapezius (LT) muscles in accordance with SENIAM recommendations.

This tooth brushing ADLs protocol (Figure 3) was performed twice: once for data acquisition with the wearable IMU–sEMG prototype device together with the Qualisys^®^ optical motion-capture system (Figure 4a), and a second time for EMG-only acquisition using the BiosignalsPlux system (Figure 4b).

First with the wearable IMU–sEMG prototype device, data acquired with the prototype device were transmitted via Bluetooth to the computer interface. EMG sensors were placed on both upper limbs following the bipolar recommendations by Surface EMG for Non-Invasive Assessment of Muscles Project (SENIAM) [42] in the same positions illustrated in our previous article [34]. After calibration, the two IMUs were positioned above the lateral epicondyle, and reference electrode placed on the olecranon [43].

Simultaneously, kinematic data were collected using the Qualisys^®^ optical motion-capture system with a total of 36 reflective markers were placed on specific anatomical landmarks of the trunk and upper limbs. For the trunk, markers were positioned on the cervical vertebra C7 (C7), thoracic vertebra T10 (T10), jugular notch (STR_UP), body of the sternum (STR_DOWN), left acromion (LAC), and right acromion (RAC). For the upper limbs, markers were placed on the left and right lateral epicondyles (LLELBOW, RLELBOW), left and right medial epicondyles (LMEELBOW, RMEELBOW), the left and right radial styloid processes (LLATWRIST, RLATWRIST), and the left and right ulnar styloid processes (LMEDWRIST, RMEDWRIST). Additional markers were positioned at the midpoint of the 3rd metacarpal on the left and right hands (L3MET, R3MET), the distal end of the 2nd metacarpal on both sides (L2MET, R2MET), and the distal end of the 5th metacarpal (L5MET, R5MET). Cluster configurations composed of four markers were placed on the anterolateral region of the left arm (LUPARM1,2,3,4), right arm (RUPARM1,2,3,4), left forearm (LARM1,2,3,4), and right forearm (RARM1,2,3,4). The 16 markers were arranged in clusters of four to track the motion of upper-limb segments, while the remaining 20 single markers were used to define the anatomical model in Visual3D (version 2025.01, C-Motion, Ontario, Canada). All recordings were preceded by system calibration.

The BiosignalsPlux^®^ acquisition followed the same protocol used with the wearable IMU–sEMG prototype device, except that—rather than two IMUs—an accelerometer was positioned above the right lateral epicondyle.

A synchronization stimulus was generated at the beginning of both acquisition procedures by tapping either the IMU (for the wearable IMU–sEMG prototype) or the accelerometer sensor (for the BiosignalsPlux^®^ acquisition). For the wearable IMU–sEMG prototype, the tap in IMU was delivered using a stick equipped with a reflective marker, which allowed the event to be captured simultaneously by the Qualisys system and the smartphone cameras. Synchronization between the IMU signals, the kinematic data acquired by the Qualisys^®^ optical motion-capture system, and the video recordings was achieved by identifying this common reference event across all three systems.

For the BiosignalsPlux^®^ acquisition, the accelerometer placed laterally above the humeral lateral epicondyle was configured to record data along the *X*-axis, and the synchronization tap was applied manually by the researcher once both the BiosignalsPlux^®^ system and smartphone cameras were recording. This impact produced a clear peak in the accelerometer signal, recorded synchronously with the EMG data and visible in the smartphone footage. This procedure ensured precise and reliable synchronization across all acquisition systems.

### 2.6. Data Analysis

To define ADL cycles, for both repetitions of the protocol (wearable IMU–sEMG prototype BiosignalsPlux^®^ acquisition), camera videos (.avi) were processed in Matlab^®^ (version R2024b; The MathWorks, Natick, MA, USA) frame-by-frame to record the start and end times of each Cycle in an Excel file, using frame rate to calculate duration, mean, and SD.

Regarding the kinematic data collected with the Qualisys^®^ optical motion-capture system, all recordings were initially processed in QTM, including one static acquisition and one dynamic acquisition corresponding to the brushing teeth ADL for each participant. During the static acquisition, reflective markers were identified, and in the dynamic acquisition their trajectories were inspected to ensure correct tracking throughout the task, starting from the moment defined as the synchronization event with the wearable IMU–sEMG prototype device. After this stage, the kinematic data were exported in .c3d format and transferred to the Visual3D software (version 2025.01) for further processing.

The biomechanical model was constructed in Visual3D by adapting an upper-limb and trunk model according to the software specifications and previously published work [44]. Segment definitions (trunk, arm, forearm, and hand) were created to establish rigid coordinate systems for each segment. Proximal and distal endpoints were identified using the 16 reflective markers placed on anatomical landmarks, complemented by 10 virtual landmarks constructed within Visual3D. An orthogonal Cartesian coordinate system was defined for each segment [41]: the *X*-axis represented the lateral (+) to medial (−) direction, the *Y*-axis the anterior (+) to posterior (−) direction, and the *Z*-axis the proximal (+) to distal (−) direction. The *X*-axis orientation differed between limbs: on right side it was oriented medially to laterally (+), whereas on left side it was oriented laterally to medially (+).

The computation of joint angles was performed in Visual3D. After applying the constructed biomechanical model to each participant’s dynamic trial, the joint angle data corresponding to the same movements and segments captured by the IMUs of the wearable IMU–sEMG prototype device were extracted. It is important to note that, for the purpose of synchronizing the data extracted from Visual3D with the data from the wearable IMU–sEMG prototype, the rotation matrix values associated with the laboratory’s Cartesian coordinate system were extracted for the left and right arm segments, rather than using the joint angle values directly computed by the software.

The EMG signals acquired by the device and by the reference system (BiosignalsPlux^®^) were processed in Matlab^®^ (version R2024b) using identical procedures. For each participant and each side, the recordings were initially trimmed based on task start and end time limits, to ensure temporal synchronization between systems. Each recording was then segmented into five movement cycles, corresponding to the five repetitions of the task. In both systems, the EMG signal was converted from digital units to volts, corrected for the mean offset, and full wave rectified. Subsequently, a 1 s moving-average filter was applied to obtain the signal envelope. Each cycle was then resampled to a fixed length of 1700 points by means of mirror padding of the signal and selection of the central window. Finally, the amplitude of each muscle was normalized between 0 and 1, for each cycle.

Next, for each participant, side, and muscle, the mean of the five normalised repetitions was computed, and the root mean square (RMS) of this mean waveform was determined. The RMS values from the device and from the BiosignalsPlux^®^ PLUX Wireless Biosignals, Lisbon, Portugal) were used in the concordance analysis, employing Bland–Altman plots (bias and 95% limits of agreement), the Pearson correlation coefficient, and the intraclass correlation coefficient ICC (2.1).

The joint orientations recorded by the IMU and by the optoelectronic reference system were processed in Matlab^®^ (version R2024b) in a harmonised manner. For each participant and each side, the recordings from both systems were trimmed using task start and end time limits, to ensure temporal correspondence between signals, and subsequently segmented into five movement cycles, corresponding to the five repetitions of the activity. The orientations measured by the IMU, provided as Euler angles (roll, pitch, yaw), were converted into 3 × 3 rotation matrices. In the optoelectronic system, the cameras provided, for each frame, rotation matrices of the segment relative to the laboratory, which were used as the basis for computing the joint angles.

First, a rigid-body transformation was estimated between the IMU coordinate system and the segment coordinate system defined by the camera model, to align the coordinate systems of the two methods. In both systems, the rotation matrices were then referenced to an initial position, defined as the mean rotation over a 0.3 s interval at the beginning of the task, and the subsequent analysis focused on relative angular variations around this reference position. From these referenced matrices, joint angles were again expressed in Euler form, unwrapped to avoid ±180° discontinuities, and smoothed using a 4th-order Butterworth low-pass filter. Finally, the IMU and camera cycles were resampled by linear interpolation to a fixed length of 1700 points, enabling point-by-point comparison over the movement cycle.

To quantify the range of motion (ROM), for each participant, side, and joint degree of freedom (3 axes), the difference between the maximum and minimum values of the relative angles obtained from the IMU and from the optoelectronic system was computed in each of the five cycles. The mean of the five repetitions yielded, for each method, one ROM value per participant, side, and joint axis. The ROM values estimated by the IMU were subsequently compared with the reference values from the cameras using concordance analysis methods, including Bland–Altman plots (bias and 95% limits of agreement), the Pearson correlation coefficient, and the intraclass correlation coefficient ICC (2.1).

**Figure 5:**
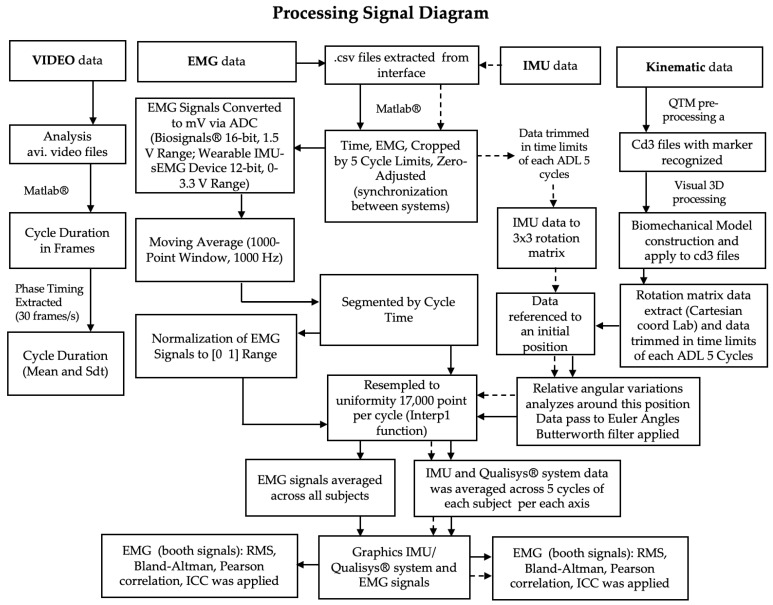
Block diagram illustrating the processing data.

A summary of the processing steps and algorithms employed is presented in the diagram below (Figure 5).

## 3. Results

The results are structured based on the signals analyzed, beginning with the presentation of findings for the EMG and then for the IMU.

### 3.1. EMG Signal

The validation analysis comparing the Wearable IMU–sEMG prototype with the BiosignalsPlux^®^ reference system revealed muscle-dependent levels of agreement across both UL. Agreement was quantified using Bland–Altman bias and Limits of Agreement (LOA), Pearson correlation coefficients, and Intraclass Correlation Coefficients (ICC), Coefficients of Variation (CV), all derived from Root Mean Square (RMS) normalized EMG signals (Table 1).

For the right UL, PM, AD, and MD demonstrated the strongest agreement between the prototype and the reference device. These muscles showed very small bias values (PM −0.03, AD +0.02 and MD +0.01), good LOA ranges, and moderate-to-strong correlations (PM r = 0.76; AD r = 0.93; MD r = 0.79). ICC values were high for all three muscles (PM 0.74, AD 0.92 and MD 0.80), supporting reliability for amplitude-based EMG analysis.

In contrast, the PD, UT, and LT exhibited low correlations (PD r = 0.28, UT r = 0.35 and LT r = 0.30) and low ICC values between devices (PD 0.27, UT 0.36, LT 0.31). Their LOA ranges were notably wider, indicating larger differences between devices.

Related to the left side, the AD and MD maintained moderate-to-strong agreement (AD r = 0.71, ICC = 0.64; MD r = 0.82, ICC = 0.79). LT and UT also demonstrated moderate agreement (LT r = 0.56, ICC = 0.54; UT r = 0.53, ICC = 0.55). Conversely, the PM on the left side showed weaker concordance (r = 0.21, ICC = 0.17), suggesting inconsistent signal reproduction for this muscle on this limb. The PD showed low agreement (r = 0.41, ICC = 0.34), consistent with their right-side results.

By analyzing the internal reliability of each system, the intra-device ICC values of the wearable IMU–sEMG prototype and the coefficients of variation (CV) calculated from the five repetitions of the ADL provide relevant information on data consistency independently of the between-method comparison.

Overall, the wearable showed moderate to high intra-device ICC values for the PM, AD, and MD muscles on both sides (ICC ranging from 0.64 to 0.92), indicating good reproducibility across consecutive task repetitions. The CV values for these muscles remained below approximately 10%, comparable to those observed for the reference system (BiosignalsPlux^®^), suggesting low and similar within-method variability. In contrast, the PD, UT, and LT muscles exhibited lower intra-device ICC values (ICC ranging from 0.24 to 0.64) and higher CVs in both systems, reflecting greater variability across repetitions.

**Figure 6:**
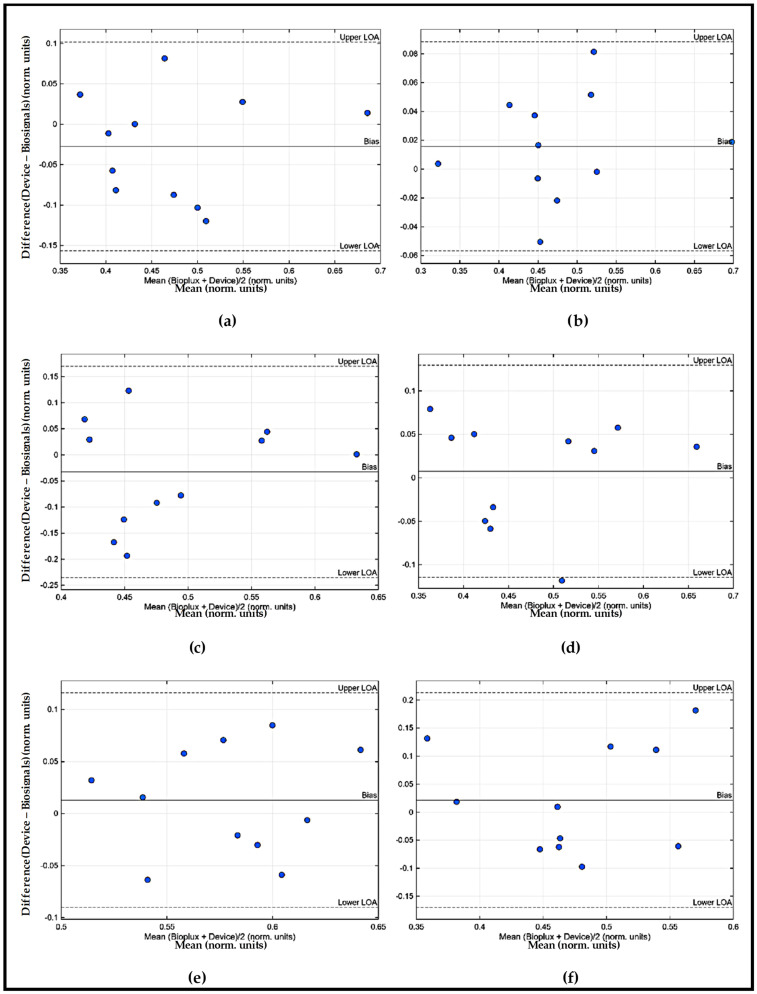
Bland–Altman RMS EMG plots of the Right UL obtained for the comparison EMG signal between the wearable IMU–sEMG prototype and the reference system: (**a**) PM; (**b**) AD; (**c**) PD; (**d**) MD; (**e**) UT; (**f**) LT.

**Figure 7:**
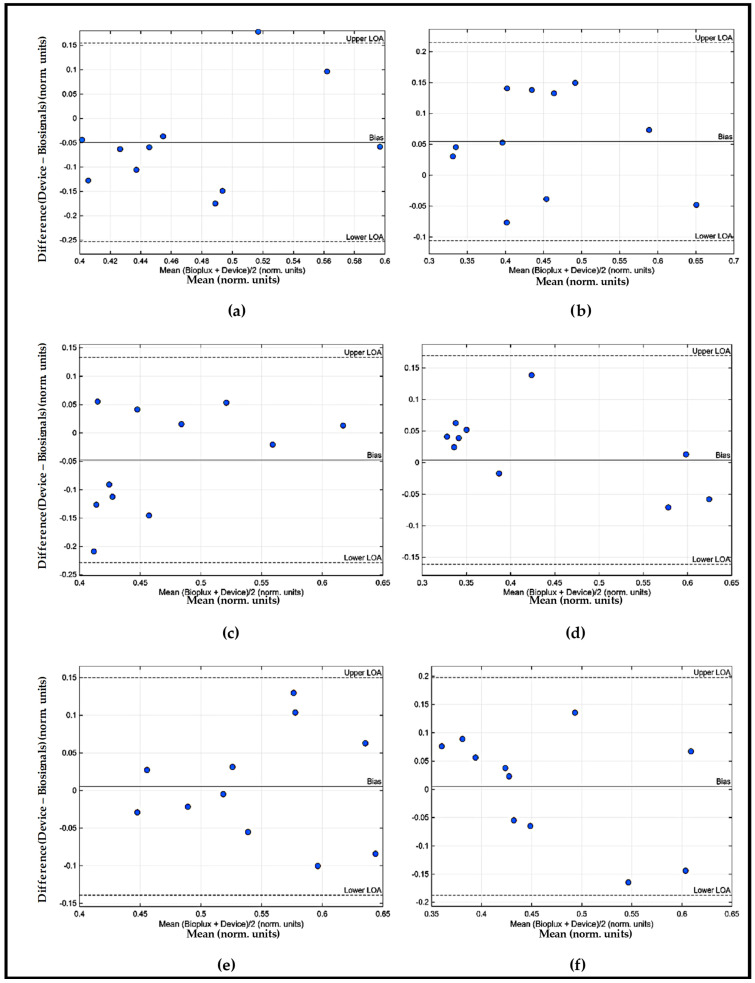
Bland–Altman RMS EMG plots of the Left UL obtained for the comparison EMG signal between the wearable IMU–sEMG prototype and the reference system: (**a**) PM; (**b**) AD; (**c**) PD; (**d**) MD; (**e**) UT; (**f**) LT.

To facilitate the interpretation of the results, Figure 6 and Figure 7 to illustrate the Bland–Altman plots obtained for the comparison between the wearable IMU–sEMG prototype and the reference system.

### 3.2. IMU Signal

The comparison between the wearable IMU–sEMG prototype and the Qualisys^®^ system revealed axis- and limb-dependent agreement across the three rotational axes of the shoulder. Agreement was quantified using Bland–Altman bias and Limits of Agreement (LOA), Pearson correlation coefficients, and Intraclass Correlation Coefficients (ICC), computed from the ROM values extracted from both systems (Table 2).

For the right UL, Axis 1 (corresponding to anterior–posterior axis and subsequently to the ABD (+)/ADD (−) movements) showed moderate agreement, with a small positive bias (+2.71°), acceptable LOA (−6.53° to 11.94°), a moderate Pearson correlation (r = 0.70), and an ICC of 0.62. For the Axis 3 (corresponding to longitudinal axis and subsequently to the RL (+)/RM (−) movements) showed similar performance, with a bias of +5.71°, LOA from −9.89° to 21.32°, and moderate correlation (r = 0.68), although ICC was lower (0.45), indicating higher variability. In contrast, Axis 2 (corresponding to medio-lateral axis and subsequently to the F (−)/E (+) movements) demonstrated poor agreement, reflected by a small bias (−2.14°) but very wide LOA (−16.98° to 12.69°), a low correlation (r = 0.18), and very low ICC (0.19). This axis showed the least reliable performance of all right-limb measures.

For the left UL, Axis 1 again showed moderate agreement (bias −3.01°, LOA −12.65° to 6.63°, r = 0.71; ICC = 0.61). Axis 3 also performed well, with bias +3.00°, LOA −8.09° to 14.09°, correlation r = 0.75, and ICC = 0.67. Notably, Axis 2 of the left UL showed the best overall agreement among all axes and limbs, with a bias of −4.75°, tighter LOA (−13.06° to +3.56°), strong correlation (r = 0.88), and the highest ICC (0.76) of the dataset—indicating that the IMU performed most consistently in this plane on the left side.

Regarding to the analysis of intra-device reliability for the kinematic measurements indicates that the wearable IMU–sEMG prototype showed moderate to high consistency across repeated executions of the ADL, depending on the movement axis and limb. For Axis 1 and Axis 3, intra-device ICC values were generally moderate to high (ICC = 0.66–0.90), with CV values below approximately 12% for the wearable and comparable values for the Qualisys^®^ system, indicating low and similar within-method variability across repetitions. In contrast, Axis 2, particularly for the right upper limb, exhibited lower intra-device ICC values and higher CVs in both systems, reflecting greater variability across repetitions.

**Figure 8:**
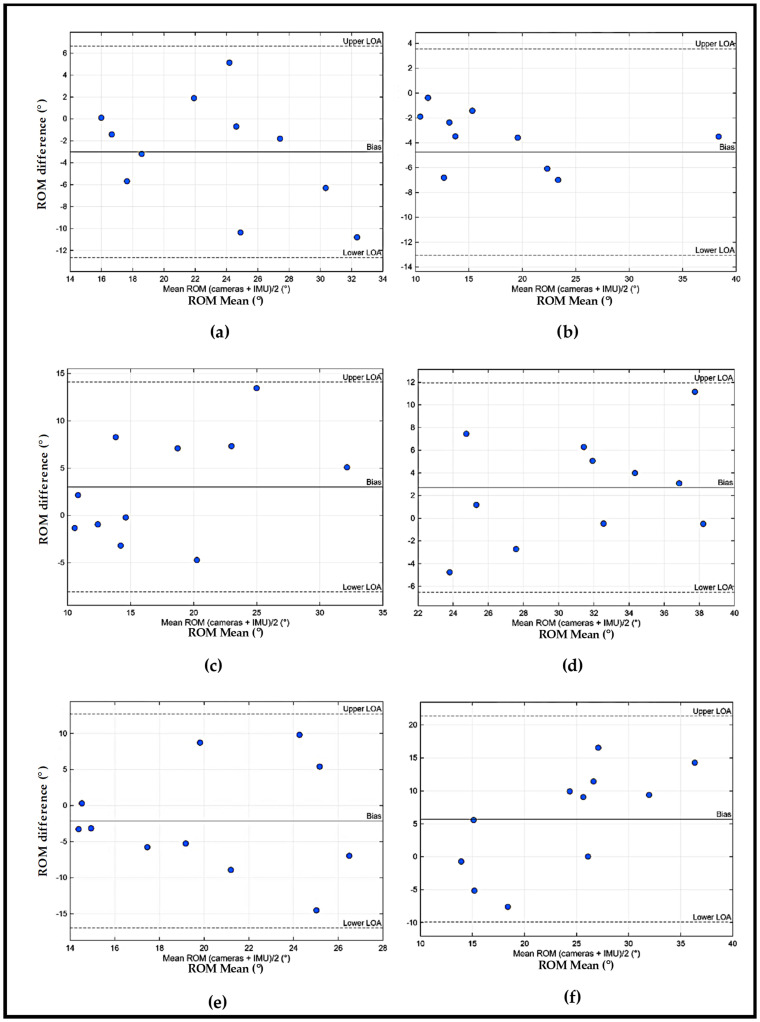
Bland–Altman ROM IMU plots obtained for the comparison Kinematic values between the wearable IMU–sEMG prototype and the reference system (Qualisys^®^ system): (**a**) Axis 1 left side (ABD/ADD); (**b**) Axis 2 left side (F/E); (**c**) Axis 3 left side (RL/RM); (**d**) Axis 1 right side (ABD/ADD); (**e**) Axis 2 right side (F/E); (**f**) Axis 3 right side (RL/RM).

To facilitate the interpretation of the results, Figure 8 illustrate the Bland–Altman plots obtained for the comparison between the wearable IMU–sEMG prototype and the reference system.

## 4. Discussion

This study aimed to validate a low-cost, wearable multimodal device integrating IMU and sEMG sensors by comparing its performance with two established gold-standard systems, BiosignalsPlux^®^ for EMG and Qualisys^®^ optical motion capture for kinematic analysis, during the execution of a bilateral brushing teeth ADL. The findings demonstrate that the proposed prototype provides measurement accuracy that is adequate for several kinematic and electrophysiological variables, though performance varies depending on the muscle examined and the motion axis considered.

Regarding EMG validation, the agreement between the prototype and the BiosignalsPlux^®^ system was muscle-dependent, a pattern consistent with previous literature on sEMG reproducibility and sensor placement sensitivity. Superficial, large-volume muscles such as the PM (right side), AD, and MD exhibited the highest concordance, as evidenced by low bias values, acceptable LOA ranges, and moderate-to-strong correlations. These muscles are characterized by favorable anatomical conditions for bipolar sensor placement (broad muscle bellies, consistent fiber orientation, and reduced subcutaneous interference) which likely contributed to the stable amplitude-based estimates across devices. The strong performance of the prototype in these muscles supports its potential use for tasks requiring gross motor evaluation or functional pattern monitoring, as often applied in rehabilitation or motor assessment studies.

Conversely, deeper or anatomically complex muscles, such as the PD, UT, LT, and PM (left side) demonstrated lower levels of agreement, with weak correlations and low ICC values. For PD, UT, LT this is consistent with reports that sEMG signal quality in these muscles is significantly affected by sensor orientation, crosstalk from adjacent musculature, and non-uniform muscle fiber recruitment [10]. For the left pectoralis major (PM), its anatomical proximity to the cardiac region may lead to interference of the EMG signal by cardiac electrical activity, which could explain the lower levels of agreement observed for the left-side PM measurements.

From an analytical and discussion perspective, the Bland–Altman plots of the EMG data (Figure 6 and Figure 7) provide important complementary information regarding the absolute agreement between the wearable IMU–sEMG prototype and the BiosignalsPlux^®^ arm movements during ADLs may introduce non-stationary biomechanical conditions that reduce stability in EMG amplitude estimation. Although this does not invalidate the prototype’s use for these muscles, it indicates the need for improved electrode design, enhanced amplification/noise reduction, or adaptive filtering techniques to ensure accurate performance in complex anatomical regions. The generally low mean bias observed across muscles indicates the absence of systematic over- or underestimation of muscle activation by the proposed device. Although some muscle-dependent variability and isolated data points outside the limits of agreement were identified, these deviations were not systematic and are consistent with the inherent variability of sEMG recordings during functional upper-limb tasks. From a clinical perspective, these results support the proposed low-cost wearable system for evaluating relative muscle activation patterns, coordination, and timing during Activities of Daily Living, which are more relevant for functional assessment and rehabilitation monitoring than absolute EMG amplitude values.

Regarding to the intra-device reliability analysis helps contextualize the agreement observed between the wearable IMU–sEMG prototype and the reference system. The moderate to high intra-device ICC values and low CVs obtained for the PM, AD, and MD muscles indicate good measurement consistency of the wearable across repeated executions of the ADL, comparable to that of the reference system. In contrast, the lower ICC values and higher CVs observed for the PD, UT, and LT muscles in both systems suggest greater intrinsic variability of EMG signals during functional task repetition, likely related to anatomical complexity and task-dependent muscle recruitment rather than to measurement instability of the wearable device.

Overall, kinematic validation of the right upper limb revealed axis-dependent agreement between the wearable IMU module and the Qualisys^®^ reference system. Bias values were generally small to moderate, indicating limited systematic error across axes. Among the evaluated axes, Axis 1 and Axis 3, corresponding, respectively, to ABD(−)/ADD(+) and RL(+)/RM(−), demonstrated acceptable agreement, characterized by moderate correlations and ICC values indicative of reasonable consistency between methods, despite increased variability for Axis 3. In contrast, Axis 2, corresponding, respectively, to F(−)/E(+), showed markedly poorer performance, with wide limits of agreement, low correlation, and poor ICC, highlighting greater variability and reduced consistency between systems for this axis. These findings indicate that while the IMU module can capture kinematic trends for certain movement components, its performance is strongly axis-dependent, particularly during complex functional tasks.

For the left upper limb, kinematic validation also revealed axis-dependent agreement between the wearable IMU module and the Qualisys^®^ reference system. Bias values ranged from −4.75° to +3.00°, indicating small to moderate systematic differences between methods. Overall, Axis 1 and Axis 3, corresponding, respectively, to ABD(−)/ADD(+) and RL(+)/RM(−), showed moderate levels of agreement, with acceptable limits of agreement and moderate Pearson correlations accompanied by ICC values suggesting reasonable consistency. In contrast, Axis 2, corresponding, respectively, to F(−)/E(+), demonstrated the strongest performance for the left limb, characterized by higher correlation and the highest ICC values, despite the presence of a moderate systematic bias. Collectively, these results indicate that kinematic agreement for the left upper limb varied across axes, with some components exhibiting greater consistency between systems than others during the execution of the functional task.

Importantly, performance differed across rotational axes. For both limbs, Axis 1 and Axis 3, yielded acceptable agreement metrics, with moderate correlations and ICC values above 0.60 in several cases. These findings suggest that the prototype can capture joint angular trends and movement trajectories with reasonable precision, an essential requirement for monitoring ADLs, evaluating rehabilitation progress, or detecting deviations in neuromotor patterns in clinical contexts. Nevertheless, Axis 2, exhibited the lowest agreement on the right upper limb, with wide LOA, low correlation, and low ICC values, and for the left upper limb, 2 showed comparatively better agreement, although consistency between methods remained limited.

Such discrepancies may arise from several factors. First, F(−)/E(+), during tooth brushing presents small angular variations compared with other movements with making it more difficult for inertial sensors to detect meaningful change. Second, soft tissue artifacts and variations in IMU alignment relative to the anatomical segment can disproportionately affect this axis. Third, bilateral task execution may introduce asymmetries in movement amplitude and coordination that are more pronounced on the dominant limb. Interestingly, Axis 2 on the left side showed the highest agreement of all axes, suggesting that symmetry or task-specific biomechanics may have influenced IMU performance in a limb-dependent manner.

For the kinematic validation, the Bland–Altman plot (Figure 7) allowed an axis-specific evaluation of agreement between the wearable IMU module and the Qualisys^®^ gold-standard system. The analysis revealed small to moderate bias values, indicating limited systematic error, while the limits of agreement highlighted axis-dependent variability associated with the biomechanical complexity of shoulder movements. Importantly, no clear magnitude-dependent trends were observed, suggesting stable IMU performance across the range of motion tested. Clinically, these findings indicate that, despite lower absolute accuracy compared to laboratory-based systems, the proposed low-cost wearable device is capable of reliably capturing joint angular trends and movement dynamics during functional ADLs, supporting its applicability for monitoring movement quality, identifying compensatory strategies, and tracking rehabilitation progress in real-world settings.

Regarding to the intra-device reliability analysis of the kinematic data provides important insight into the sources of agreement and disagreement observed between the wearable IMU–sEMG prototype and the optoelectronic reference system. The moderate to high intra-device ICC values and comparable CVs observed for Axis 1 and Axis 3 indicate that the wearable IMU yields consistent ROM estimates across repeated executions of the ADL, supporting the stability of its measurements for these movement components. In contrast, the lower ICC values and higher CVs observed for Axis 2, particularly in the right upper limb, suggest greater intrinsic variability associated with this movement during task repetition. As similar variability patterns were observed in the Qualisys^®^ system, the reduced inter-method agreement for this axis is more likely attributable to task-dependent biomechanics and movement complexity rather than to measurement noise or instability of the wearable device.

With respect to the metrics reported for other devices [11,15], an evident limitation can be identified, as there are no comprehensive validation data available for fully integrated systems combining IMU and sEMG technologies. In the study that reports the use of these two technologies in an integrated manner [11], validation results are provided primarily for the IMU component, with limited validation of the sEMG module. When compared with the Knee Angle Measurement (KAM) system [11], which demonstrated very high kinematic accuracy under controlled conditions and during lower-limb gait analysis, the present wearable IMU–sEMG prototype showed lower absolute kinematic accuracy during upper-limb ADLs. However, this comparison must be interpreted considering the greater biomechanical complexity addressed in the present study, as the wearable IMU–sEMG prototype was evaluated during multi-planar shoulder movements performed in functional tasks, which pose substantially higher challenges for wearable kinematic assessment. Importantly, unlike the KAM system, the proposed device enables bilateral upper-limb assessment and synchronized acquisition of kinematic and sEMG data during functional activities, extending its applicability beyond single-joint or unilateral analyses. These features represent a significant advantage for neuromotor pattern analysis and functional assessment in real-world and clinical contexts, where task complexity and bilateral coordination are essential.

Compared with studies that combined kinematic and biosignal acquisition using separate devices operating in parallel, such as MPU6050-based IMUs coupled with a Wave COMETA multi-channel EMG system [15], the present wearable IMU–sEMG prototype also differentiates itself by adopting an integrated approach to synchronized data acquisition. While the study by [16] reported very strong IMU validity against a VICON gold standard, with low bias (≈0.22°), narrow limits of agreement, and high concordance and linearity, this performance was achieved using distinct acquisition systems and under controlled validation conditions. In contrast, the present study evaluated an integrated, low-cost wearable device during complex upper-limb ADLs, including bilateral movements, resulting in moderate but task-consistent kinematic agreement. Furthermore, unlike [15], the EMG component in the present study was quantitatively validated against a commercial reference system, enabling a more comprehensive assessment of muscle activation agreement. These differences highlight a trade-off between maximal kinematic accuracy obtained with parallel high-end systems and the functional applicability, integration, and bilateral assessment capabilities offered by the proposed device.

Taken together, the results highlight that the proposed Wearable IMU–sEMG prototype is capable of reliably estimating kinematic and EMG characteristics for a relevant subset of muscles and movement directions commonly involved in ADLs.

From a broader perspective, this study contributes to the ongoing development of affordable, portable, and integrated sensing solutions that facilitate quantitative movement analysis outside controlled laboratory environments. Such technologies are critical in supporting continuous rehabilitation monitoring, early detection of compensatory behaviors, and personalized therapeutic interventions for individuals with neurological impairments. Although the present prototype shows promising potential, targeted refinements, such as improved calibration routines, optimized IMU housing, enhanced EMG electrode adhesion, or advanced signal fusion algorithms, may further strengthen its reliability across all variables and expand its applicability to other ADLs or patients.

Future research should include larger and more diverse samples, exploration of additional ADLs with different biomechanical requirements, and validation in clinical populations with neuromotor deficits, where movement variability and compensatory strategies are more pronounced. Integrating machine learning-based classification approaches to automatically detect atypical movement patterns from multimodal data streams may further enhance clinical utility.

## 5. Conclusions

The findings of this study support the validity of a low-cost, portable multimodal wearable device capable of acquiring synchronized IMU and sEMG signals during the execution of functional activities of daily living (ADLs). ADLs. The device’s performance was benchmarked against two established gold-standard systems, BiosignalsPlux^®^ for EMG and Qualisys^®^ optical motion capture for kinematic measurements, demonstrating that the prototype provides moderate-to-strong agreement in several clinically relevant variables.

For EMG, the device showed good reliability in superficial shoulder muscles (PM, AD, MD) and moderate performance in deeper or anatomically complex muscles (PD, UT, LT). For kinematic measurements, the IMU sensors achieved adequate validity in flexion–extension and internal–external rotation, with the best results observed in left-limb movements. However, performance in abduction–adduction, particularly on the dominant limb, remains an area for improvement.

Overall, these findings indicate that the proposed device is suitable for functional monitoring of neuromotor patterns during ADLs, with strengths in capturing global movement patterns and muscle activation amplitudes relevant to rehabilitation and motor assessment. Its low cost, portability, and bilateral acquisition capability represent important advantages over existing systems, supporting its potential adoption in clinical, research, and home-based rehabilitation settings.

Nonetheless, optimization of the sensors, calibration routines, and processing algorithms is recommended to enhance performance in anatomically challenging muscles and motion axes. Future studies involving stroke survivors or other clinical populations will be essential to fully establish the device’s capacity for detecting compensatory behaviors, quantifying impairment, and monitoring rehabilitation outcomes.

In conclusion, the validated IMU–sEMG prototype constitutes a promising step toward accessible and scalable multimodal monitoring technologies capable of supporting quantitative assessment of upper-limb motor function during ADLs, ultimately contributing to more personalized and effective rehabilitation strategies.

## Figures and Tables

**Table 1:** EMG validation metrics comparing the wearable IMU–sEMG prototype with the BiosignalsPlux^®^ reference system for both upper limbs.

Muscle	Limb	Bias	LOA (Lower–Upper)	Pearson r	ICC (Between Devices)	ICC (Wearable IMU–sEMG Prototype)	CV (%) (Wearable IMU–sEMG Prototype)	CV (%) (Biosignals)
PM	Right	−0.03	−0.16 to 0.10	0.76	0.74	0.82	10.56	6.90
	Left	−0.05	−0.25 to 0.15	0.21	0.17	0.20	9.91	8.43
AD	Right	+0.02	−0.06 to 0.09	0.93	0.92	0.92	0.92	7.59
	Left	+0.05	−0.11 to 0.21	0.71	0.64	0.64	8.58	6.76
MD	Right	+0.01	−0.11 to 0.13	0.79	0.80	0.78	9.67	8.45
	Left	+0.00	−0.16 to 0.17	0.82	0.79	0.72	10.20	7.21
PD	Right	−0.03	−0.24 to 0.17	0.28	0.27	0.24	11.70	11.16
	Left	−0.05	−0.23 to 0.13	0.41	0.34	0.41	10.60	8.82
UT	Right	+0.01	−0.09 to 0.12	0.35	0.36	0.41	6.25	6.78
	Left	+0.01	−0.14 to 0.15	0.53	0.55	0.64	8.10	7.27
LT	Right	+0.02	−0.17 to 0.21	0.30	0.31	0.29	8.77	8.41
	Left	+0.01	−0.19 to 0.20	0.56	0.54	0.58	12.17	9.59

**Table 2:** Kinematic validation metrics comparing the wearable IMU–sEMG prototype with the Qualisys^®^ reference system in both upper limbs.

Limb	Axis	Bias (°)	LOA (Lower– Upper)	Pearson r	ICC (Between Devices)	ICC (Wearable IMU–sEMG Prototype)	CV (%) (Wearable IMU–sEMG Prototype)	CV (%) (Qualisys^®^ System)
Right	Axis 1	2.71	−6.53 to 11.94	0.70	0.62	0.83	7.20	7.98
Right	Axis 2	−2.14	−16.98 to 12.69	0.18	0.19	0.42	14.23	10.12
Right	Axis 3	5.71	−9.89 to 21.32	0.68	0.45	0.66	11.17	11.72
Left	Axis 1	−3.01	−12.65 to 6.63	0.71	0.61	0.90	15.59	13.46
Left	Axis 2	−4.75	−13.06 to 3.56	0.88	0.76	0.00	16.53	15.20
Left	Axis 3	3.00	−8.09 to 14.09	0.75	0.67	0.86	17.75	16.56

## Data Availability

The original contributions presented in this study are included in the article. Further inquiries can be directed to the corresponding author.
